# Estimated glucose disposal rate shows the broadest associations with cardiometabolic multimorbidity progression in early cardiovascular–kidney–metabolic stages: a longitudinal cohort study

**DOI:** 10.3389/fendo.2026.1948249

**Published:** 2026-09-30

**Authors:** Zunwei Wang, Yanyan Liu, Yitong Ling, Yujie Sun

**Affiliations:** 1 Department of Neurology, The Affiliated Hospital of Qingdao University, Qingdao, China; 2 Department of Gastroenterology, The Affiliated Hospital of Qingdao University, Qingdao, China; 3 Qingdao Medical College, Qingdao University, Qingdao, China

**Keywords:** atherogenic index of plasma, cardiometabolic multimorbidity, cardiovascular–kidney–metabolic syndrome, estimated glucose disposal rate, insulin resistance, risk prediction, triglyceride–glucose index

## Abstract

**Background:**

Early risk stratification may help prevent cardiometabolic multimorbidity (CMM) among adults at cardiovascular–kidney–metabolic (CKM) stages 0–3. We compared the estimated glucose disposal rate (eGDR), triglyceride–glucose (TyG) index, and atherogenic index of plasma (AIP) for incident CMM, disease transitions, and incremental prediction.

**Methods:**

This longitudinal cohort analysis included 6,407 China Health and Retirement Longitudinal Study participants aged 45–79 years at CKM stages 0–3 and free of CMM in 2011, with follow-up through 2020. CMM was defined as the coexistence of at least two of diabetes, heart disease, and stroke. Cox models estimated hazard ratios (HRs) per standard deviation in the adverse direction (lower eGDR or higher TyG or AIP). Restricted cubic-spline and multistate models assessed dose–response patterns and transitions; five repetitions of stratified fivefold out-of-fold validation evaluated incremental 9-year prediction.

**Results:**

During a median follow-up of 9.0 years, 593 participants developed CMM. Primary-model HRs per adverse standard deviation were 1.92 (95% confidence interval [CI], 1.77–2.09) for eGDR, 1.69 (95% CI, 1.57–1.82) for TyG, and 1.51 (95% CI, 1.39–1.64) for AIP. After CKM-stage adjustment, eGDR remained associated with all three disease-accumulation transitions (false-discovery-rate-adjusted P ≤ 0.009); TyG remained associated only with the earliest transition, and no AIP transition survived correction. Among single-marker additions, eGDR yielded the largest point gain in discrimination (ΔC-index, 0.041; 95% CI, 0.027–0.055). Adding TyG to eGDR further increased the C-index by 0.012 (95% CI, 0.005–0.020), whereas adding AIP to eGDR plus TyG changed the C-index by 0.001 (95% CI, −0.003 to 0.005). Calibration slopes were 0.910–0.945. Interval-aware and competing-risk analyses were consistent.

**Conclusion:**

All three indices were associated with incident CMM. eGDR showed the most consistent transition associations and the numerically largest single-marker gain in internally validated prediction; TyG was complementary, whereas AIP added little beyond eGDR and TyG. Independent external validation and clinical-utility assessment are needed before use in early risk stratification.

## 1 Introduction

Cardiovascular-kidney-metabolic (CKM) syndrome brings obesity, abnormal glucose metabolism, dyslipidemia, hypertension, chronic kidney disease, and cardiovascular disease into a single prevention-oriented framework (1, 2). At CKM stages 0–3, individuals have not yet developed overt cardiovascular disease, although cardiometabolic risk may already be measurable. Risk stratification during these stages could identify high-risk individuals earlier and support timely, more intensive prevention.

Cardiometabolic multimorbidity (CMM), generally defined as the coexistence of at least two major cardiometabolic diseases, is a particularly important outcome in aging populations. CMM is associated with markedly higher mortality than individual cardiometabolic diseases (3). Multimorbidity more broadly is also associated with poorer function, reduced quality of life, and greater healthcare use (4). For middle-aged and older adults at early CKM stages, preventing CMM may have more clinical and public health value than focusing on diabetes, heart disease, or stroke separately.

Insulin resistance and atherogenic dyslipidemia may link early CKM to the later development of CMM. Both can be assessed using indices derived from routine clinical data. The estimated glucose disposal rate (eGDR) combines waist circumference, hypertension status, and glycated hemoglobin (HbA1c) and serves as a practical surrogate marker of insulin resistance (5). The triglyceride–glucose (TyG) index uses fasting glucose and triglyceride levels and is commonly used to estimate insulin resistance without measuring insulin (6). Unlike the other two indices, the atherogenic index of plasma (AIP) reflects atherogenic dyslipidemia and is calculated as log_10_(triglycerides/high-density lipoprotein cholesterol) (7). Because all three rely on routine clinical measures, they can be calculated at low cost and applied reproducibly in large population cohorts.

Previous CHARLS research linked the TyG index to incident CMM (8). Two recent CHARLS analyses directly compared multiple insulin-resistance surrogates and identified eGDR as the strongest discriminator of CMM (9, 10). Related CHARLS studies have examined these indices in relation to cardiovascular disease, stroke, mortality, and CKM progression (11–16). Existing studies have not jointly compared eGDR, TyG, and AIP within a fixed CMM-free early-CKM cohort across incident CMM, transition-specific disease accumulation, and mutually conditional incremental prediction using all eight marker combinations. Whether TyG or AIP contributes prognostic information beyond eGDR therefore remains uncertain.

We therefore used longitudinal CHARLS data to compare eGDR, TyG, and AIP among adults at CKM stages 0–3 who were free of CMM at baseline. We examined their associations with incident CMM, characterized transition-specific disease accumulation, and quantified the incremental prognostic information provided by all eight marker combinations under repeated out-of-fold validation.

## 2 Materials and methods

### 2.1 Study design and participants

We conducted a longitudinal cohort study using prospective China Health and Retirement Longitudinal Study (CHARLS) data, with the 2011 interview as baseline and the 2013, 2015, 2018, and 2020 surveys as follow-up. CHARLS enrolled community-dwelling Chinese adults aged 45 years or older through multistage probability sampling across 28 provinces (17). The Biomedical Ethics Review Committee of Peking University approved the protocol (IRB00001052–11015), and all participants provided written informed consent. We analyzed de-identified data.

Eligible participants were aged 45–79 years, had fasted before blood collection, and had valid values for all three indices. They also required determinate absence of baseline heart disease and stroke, at least one determinate CMM follow-up assessment, a classifiable baseline CKM stage of 0–3, and complete data for all model covariates. No formal sample-size calculation was performed; all participants meeting the prespecified eligibility criteria were included. **Figure 1** shows the selection process.

**Figure 1:**
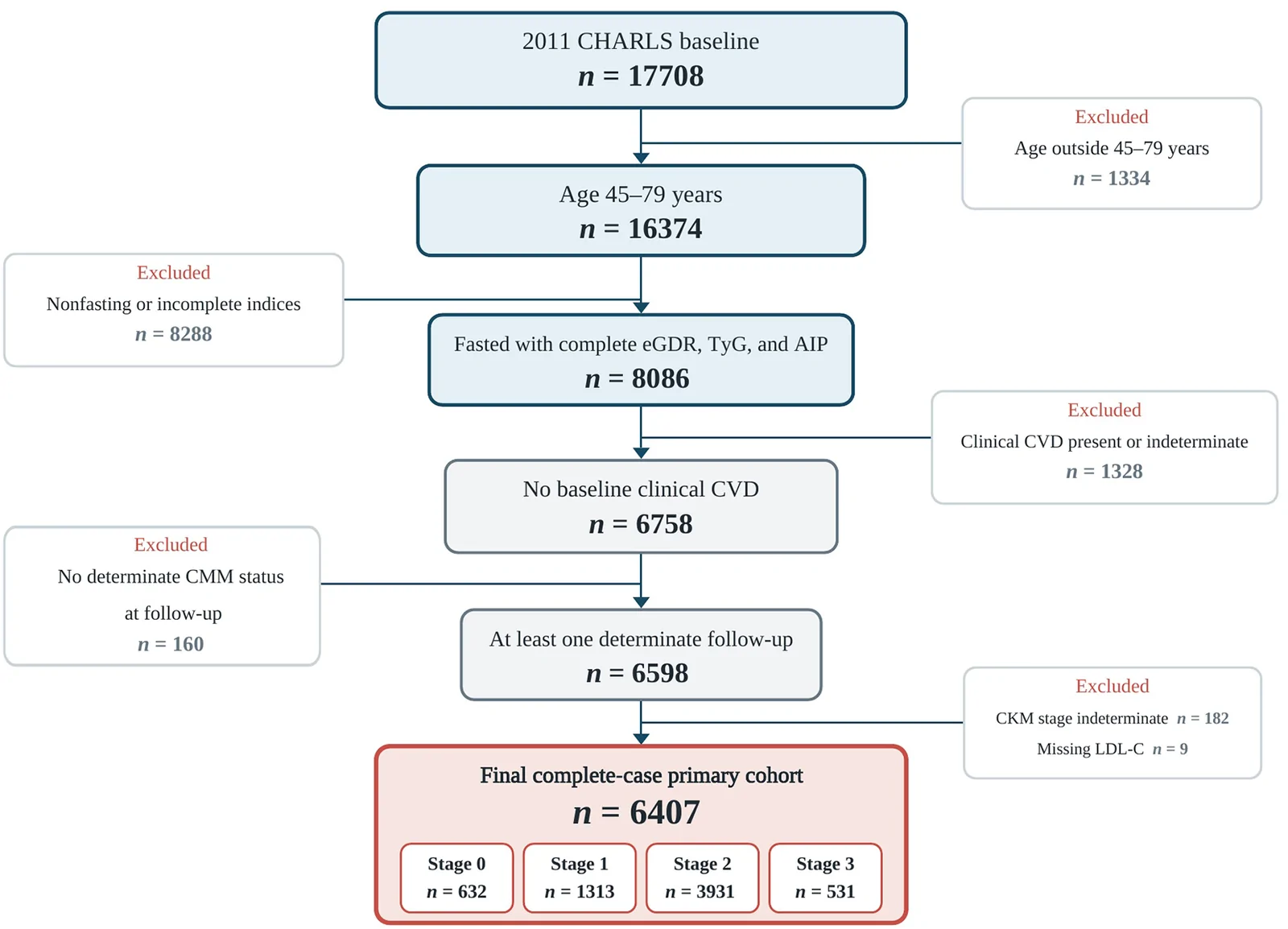
Selection of the final complete-case primary cohort. Sequential selection of the primary cohort from the 2011 CHARLS baseline. Exclusions were applied sequentially, resulting in 6,407 participants with CKM stages 0–3. Among the 160 participants without a determinate CMM status at any follow-up wave, 155 had no recorded follow-up participation, whereas five participated in at least one follow-up wave but had insufficient disease information to determine CMM status. AIP, atherogenic index of plasma; CHARLS, China Health and Retirement Longitudinal Study; CKM, cardiovascular–kidney–metabolic; CMM, cardiometabolic multimorbidity; CVD, cardiovascular disease; eGDR, estimated glucose disposal rate; LDL-C, low-density lipoprotein cholesterol; TyG, triglyceride–glucose index.

### 2.2 Exposures and CKM staging

All indices used 2011 measurements. eGDR was calculated as 21.158 − 0.09 × waist circumference (cm) − 3.407 × hypertension − 0.551 × HbA1c (%). For the eGDR calculation, hypertension was defined as systolic blood pressure ≥140 mmHg or diastolic blood pressure ≥90 mmHg, or documented diagnosis or antihypertensive treatment, consistent with the established eGDR definition (5). For CKM-stage assignment, hypertension was defined separately as systolic blood pressure ≥130 mmHg or diastolic blood pressure ≥80 mmHg, or diagnosis or treatment evidence, consistent with the CKM staging criteria (2). TyG was calculated as ln[fasting triglycerides (mg/dL) × fasting plasma glucose (mg/dL)/2], and AIP as log_10_[triglycerides (mmol/L)/HDL cholesterol (mmol/L)] (6, 7). eGDR was multiplied by −1 before standardization. All indices were standardized in the stage-determinate source cohort before complete-case restriction. HRs therefore represent a 1-SD adverse difference. Measurement and conversion details are provided in **Supplementary Material 1**.

CKM stage followed a CHARLS-adapted operationalization of the 2026 AHA/ACC/ADA/ASN guideline (2). Stage 1 comprised excess or dysfunctional adiposity or prediabetes. Stage 2 comprised hypertension, diabetes, fasting triglycerides ≥150 mg/dL, metabolic syndrome, or chronic kidney disease. Stage 3 required eGFR <30 mL/min/1.73 m² or 10-year total-CVD risk ≥20% from the base PREVENT equation (18). eGFR used the 2021 race-free CKD-EPI creatinine equation (19). Baseline heart disease or stroke indicated stage 4 and was excluded. Stages were assigned from high to low without treating missing evidence as absence (**Table 1**).

**Table 1:** Operational definition of baseline CKM stages in CHARLS.

CKM stage	Study-specific operational definition
Stage 0	No positive criterion for stages 1–3, with all relevant evidence observed and negative.
Stage 1	BMI ≥23 kg/m², central obesity (waist circumference ≥90 cm in men or ≥80 cm in women), or prediabetes, with no criterion for a higher stage.
Stage 2	At least one of the following: hypertension (systolic blood pressure ≥130 mmHg or diastolic blood pressure ≥80 mmHg, or a self-reported physician diagnosis or antihypertensive medication use), diabetes, fasting triglycerides ≥1.7 mmol/L (150 mg/dL), metabolic syndrome, or chronic kidney disease (eGFR <60 mL/min/1.73 m² or kidney-disease history), with no criterion for stage 3 or clinical cardiovascular disease.
Stage 3	eGFR <30 mL/min/1.73 m² or 10-year total cardiovascular disease risk ≥20% estimated with the base PREVENT equation, without baseline clinical cardiovascular disease.
Stage 4	Baseline heart disease or stroke identified from diagnosis or disease-specific treatment evidence; excluded from the analytic cohort.
Indeterminate	Evidence needed to establish the highest stage was missing. Missing evidence was not treated as absence; these participants were excluded from the analytic cohort.

Stages were assigned from highest to lowest. Prediabetes was defined as fasting plasma glucose 5.6–6.9 mmol/L (100–125 mg/dL) or HbA1c 5.7%–6.4% without diabetes. Diabetes was defined by diagnosis/treatment, fasting plasma glucose ≥7.0 mmol/L (126 mg/dL), or HbA1c ≥6.5%. Metabolic syndrome required at least three of central obesity, triglycerides ≥1.7 mmol/L (150 mg/dL), low HDL-C (<1.0 mmol/L [40 mg/dL] in men or <1.3 mmol/L [50 mg/dL] in women), elevated blood pressure or diagnosis/treatment, and fasting plasma glucose ≥5.6 mmol/L (100 mg/dL) or diabetes. BMI, body mass index; CHARLS, China Health and Retirement Longitudinal Study; CKM, cardiovascular–kidney–metabolic; eGFR, estimated glomerular filtration rate; HbA1c, glycated hemoglobin; HDL-C, high-density lipoprotein cholesterol; PREVENT, Predicting Risk of Cardiovascular Disease EVENTs.

### 2.3 Outcome and covariates

Incident CMM was the cumulative coexistence of at least two of diabetes, heart disease, and stroke (3, 8). From 2011–2018, each condition required a physician diagnosis or disease-specific medication. Baseline diabetes also included fasting plasma glucose ≥126 mg/dL or HbA1c ≥6.5%. Because comparable medication items were unavailable in 2020, new 2020 cases relied on diagnosis; previous disease histories were carried forward. Event time was the midpoint between the last determinate CMM-negative and first positive surveys. Participants without CMM were censored at their last determinate negative survey. **Supplementary Material 1** details the tri-state disease rules and event-time sensitivity analysis.

### 2.4 Statistical analysis

Continuous variables were summarized as median (interquartile range) and categorical variables as n (%). Pairwise correlations among eGDR, TyG, and AIP were summarized using Spearman rank correlation coefficients. Cox models used time since the 2011 interview and Efron handling of ties. Model 1 adjusted for age and sex, which were prespecified as the minimum adjustment set. Model 2 added education, rural residence, marital status, current smoking, and past-year alcohol use. The primary Model 3 added eGFR, LDL cholesterol, ln(C-reactive protein + 0.1), and uric acid. Model 4 added baseline CKM stage as a sensitivity analysis because of metabolic overlap. We reported HRs with 95% CIs and assessed proportional hazards using Schoenfeld residuals.

Indices were modeled continuously and in adverse-direction quartiles; Q4 represented the lowest eGDR or highest TyG or AIP. Trend tests used within-quartile medians. Restricted cubic splines used knots at the 5th, 27.5th, 50th, 72.5th, and 95th percentiles, with the median as reference. Curves were displayed from the 1st to 99th percentiles. Survey weights were omitted because the estimand was a within-cohort association, not a population-representative effect.

Sensitivity analyses included interval-aware discrete-time models, Cox models excluding participants with baseline diabetes, competing-risk models for death before CMM (20), and Cox models restricted to follow-up through 2018. Model 3 was primary, whereas Model 4 additionally adjusted for baseline CKM stage.

Exploratory multistate analyses examined transitions among no CMM component, one component, CMM, and death before CMM (21). Each index was modeled under Models 3 and 4. Prediction analyses compared the base model with all seven index combinations for 9-year CMM risk using repeated out-of-fold validation and established measures of discrimination, calibration, and prediction error for censored outcomes (22–27).

Tests were two-sided with P < 0.05. Multiplicity adjustment was confined to the sensitivity, multistate, and prediction families described above. No missing baseline exposure or model covariate values were imputed; missing follow-up disease states were coded as unknown and handled according to the risk-set rules for each analysis. Analyses used R 4.5.2 and Python 3.14.3. Detailed statistical methods are provided in **Supplementary Material 1**.

## 3 Results

### 3.1 Study population and incident CMM

Of 17,708 baseline participants, 16,374 were aged 45–79 years, 8,086 had fasted and had complete data for eGDR, TyG, and AIP, and 6,758 had determinate absence of baseline heart disease and stroke. Among these 6,758 participants, 155 (2.3%) had no recorded participation at any follow-up wave, whereas five attended at least one follow-up wave but had insufficient disease information to determine CMM status. Thus, 160 participants (2.4%) had no determinate CMM status at any follow-up wave and were excluded, leaving 6,598 participants with at least one determinate follow-up assessment. After excluding 182 participants with an indeterminate baseline CKM stage, 6,416 remained; nine (0.14%) were subsequently excluded because of missing LDL-C, leaving 6,407 participants in the final complete-case cohort (**Figure 1**).

Median age was 57 years (interquartile range, 51–64); 3,041 (47.5%) were men and 4,218 (65.8%) lived in rural areas. CKM stages 0, 1, 2, and 3 included 632, 1,313, 3,931, and 531 participants, respectively (**Table 2**). Baseline characteristics were generally similar between included and excluded participants, with modest differences in rural residence, education, and waist circumference (maximum absolute standardized mean difference [SMD], 0.180; **Supplementary Material 2**; **Supplementary Table S5**). At baseline, eGDR was inversely correlated with TyG (Spearman ρ = −0.332) and AIP (ρ = −0.304), whereas TyG and AIP were strongly positively correlated (ρ = 0.891; all P < 0.001; **Supplementary Material 2**; **Supplementary Table S6**).

**Table 2:** Baseline characteristics of the complete-case primary cohort.

Characteristic	Overall (N = 6,407)	CMM-free (n=5,814)	Incident CMM (n=593)	P value
Demographic and lifestyle characteristics				
Age, years	57.00 (51.00–64.00)	57.00 (51.00–64.00)	59.00 (54.00–65.00)	<0.001
Male	3041 (47.5%)	2792 (48.0%)	249 (42.0%)	0.005
Rural residence	4218 (65.8%)	3844 (66.1%)	374 (63.1%)	0.136
Married or partnered	5741 (89.6%)	5217 (89.7%)	524 (88.4%)	0.299
Current smoking	2013 (31.4%)	1861 (32.0%)	152 (25.6%)	0.001
Alcohol use in the past year	2206 (34.4%)	2021 (34.8%)	185 (31.2%)	0.082
Anthropometric and clinical characteristics				
Body mass index, kg/m²	23.07 (20.80–25.60)	22.92 (20.69–25.39)	24.90 (22.27–27.45)	<0.001
Waist circumference, cm	84.20 (77.80–91.50)	84.00 (77.20–91.00)	90.00 (82.80–97.00)	<0.001
Systolic blood pressure, mmHg	125.50 (113.00–140.00)	124.50 (112.50–139.00)	133.50 (120.50–150.00)	<0.001
Baseline diabetes	943 (14.7%)	678 (11.7%)	265 (44.7%)	<0.001
Hypertension (eGDR definition)	2382 (37.2%)	2029 (34.9%)	353 (59.5%)	<0.001
Chronic kidney disease	388 (6.1%)	331 (5.7%)	57 (9.6%)	<0.001
Laboratory and metabolic characteristics				
Fasting plasma glucose, mg/dL	101.88 (94.14–111.96)	101.34 (93.96–110.16)	113.40 (99.90–138.42)	<0.001
HbA1c, %	5.10 (4.90–5.40)	5.10 (4.90–5.40)	5.30 (5.00–5.90)	<0.001
Triglycerides, mg/dL	102.66 (73.45–148.68)	100.89 (71.69–145.14)	124.78 (89.39–187.62)	<0.001
HDL-C, mg/dL	49.87 (40.98–60.31)	50.26 (41.37–61.08)	45.62 (37.11–54.90)	<0.001
LDL-C, mg/dL	114.82 (93.94–137.82)	114.43 (93.94–136.86)	119.07 (97.04–145.36)	<0.001
eGFR, mL/min/1.73 m²	99.71 (90.29–106.21)	99.94 (90.57–106.51)	98.06 (88.38–103.96)	<0.001
Estimated glucose disposal rate, mg/kg/min	10.06 (7.40–11.14)	10.20 (7.60–11.20)	7.40 (6.13–9.97)	<0.001
Triglyceride-glucose index	8.56 (8.20–9.00)	8.53 (8.18–8.95)	8.89 (8.46–9.47)	<0.001
Atherogenic index of plasma	−0.05 (−0.26 to 0.17)	−0.07 (−0.27 to 0.16)	0.08 (−0.13 to 0.31)	<0.001
Baseline CKM stage				
Stage 0	632 (9.9%)	620 (10.7%)	12 (2.0%)	<0.001
Stage 1	1313 (20.5%)	1264 (21.7%)	49 (8.3%)	
Stage 2	3931 (61.4%)	3491 (60.0%)	440 (74.2%)	
Stage 3	531 (8.3%)	439 (7.6%)	92 (15.5%)	

Values are median (interquartile range) or n (%). Available n was 6,368 for body mass index and 6,398 for chronic kidney disease; all other characteristics had n = 6,407. P values compare participants with and without incident CMM and were calculated using the Wilcoxon rank-sum test for continuous variables and Pearson’s chi-square test without continuity correction for categorical variables. The P value for CKM stage is an overall test across stages 0–3 and is shown once. Hypertension in this table uses the eGDR definition (blood pressure ≥140/90 mmHg or diagnosis/treatment); CKM staging used ≥130/80 mmHg or diagnosis/treatment. CKM, cardiovascular–kidney–metabolic; CMM, cardiometabolic multimorbidity; eGDR, estimated glucose disposal rate; eGFR, estimated glomerular filtration rate; HbA1c, glycated hemoglobin; HDL-C, high-density lipoprotein cholesterol; LDL-C, low-density lipoprotein cholesterol.

Over a median 9.0 years (interquartile range 8.8–9.0), 593 CMM events occurred during 51,528 person-years. The incidence rate was 11.5 per 1000 person-years (95% CI 10.6–12.5). The proportion with incident CMM ranged from 1.9% in stage 0 to 17.3% in stage 3.

### 3.2 Associations of eGDR, TyG, and AIP with incident CMM

In primary Model 3, HRs were 1.92 (95% CI 1.77–2.09) per 1-SD lower eGDR and 1.69 (95% CI 1.57–1.82) per 1-SD higher TyG. The HR for higher AIP was 1.51 (95% CI 1.39–1.64). All P values were <0.001. For adverse-direction Q4 versus Q1, Model 3 HRs were 6.30 (95% CI 4.60–8.64) for eGDR and 3.65 (95% CI 2.79–4.77) for TyG. The AIP HR was 2.60 (95% CI 2.01–3.36) (**Table 3**). All trend tests gave P < 0.001. Restricted cubic splines indicated nonlinearity for eGDR (P < 0.001), but not TyG (P = 0.649) or AIP (P = 0.508) (**Figure 2**).

**Table 3:** Associations of eGDR, TyG, and AIP with incident CMM in sequential Cox models.

Index and contrast	N/events	Model 1 HR (95% CI)	Model 2 HR (95% CI)	Model 3 HR (95% CI)	Model 4 (+ CKM stage) HR (95% CI)
eGDR (lower values adverse)					
Per 1-SD lower	6407/593	1.98 (1.83–2.14)	1.97 (1.82–2.14)	1.92 (1.77–2.09)	1.68 (1.53–1.85)
Q1 (≥11.142400; reference)	1603/47	Reference	Reference	Reference	Reference
Q2 (10.058700 to <11.142400)	1601/94	2.05 (1.45–2.91)	2.04 (1.44–2.90)	1.97 (1.39–2.80)	1.76 (1.22–2.53)
Q3 (7.398350 to <10.058700)	1601/156	3.48 (2.51–4.82)	3.47 (2.50–4.81)	3.35 (2.41–4.65)	2.37 (1.67–3.37)
Q4 (<7.398350)	1602/296	6.85 (5.03–9.32)	6.78 (4.97–9.25)	6.30 (4.60–8.64)	4.04 (2.86–5.71)
P for trend	6407/593	<0.001	<0.001	<0.001	<0.001
TyG (higher values adverse)					
Per 1-SD higher	6407/593	1.66 (1.55–1.77)	1.65 (1.54–1.77)	1.69 (1.57–1.82)	1.48 (1.37–1.61)
Q1 (≤8.202984; reference)	1602/72	Reference	Reference	Reference	Reference
Q2 (>8.202984 to ≤8.563289)	1602/109	1.49 (1.10–2.00)	1.48 (1.10–2.00)	1.44 (1.07–1.94)	1.29 (0.96–1.75)
Q3 (>8.563289 to ≤8.998528)	1601/150	2.09 (1.58–2.77)	2.08 (1.56–2.75)	1.96 (1.47–2.61)	1.54 (1.15–2.06)
Q4 (>8.998528)	1602/262	3.90 (3.00–5.06)	3.85 (2.96–5.00)	3.65 (2.79–4.77)	2.36 (1.79–3.12)
P for trend	6407/593	<0.001	<0.001	<0.001	<0.001
AIP (higher values adverse)					
Per 1-SD higher	6407/593	1.48 (1.37–1.59)	1.47 (1.36–1.58)	1.51 (1.39–1.64)	1.30 (1.19–1.42)
Q1 (≤−0.255504; reference)	1603/84	Reference	Reference	Reference	Reference
Q2 (>−0.255504 to ≤−0.053916)	1602/119	1.43 (1.09–1.90)	1.43 (1.08–1.90)	1.37 (1.03–1.81)	1.25 (0.94–1.66)
Q3 (>−0.053916 to ≤0.171517)	1601/169	2.07 (1.59–2.69)	2.05 (1.58–2.67)	1.90 (1.45–2.47)	1.51 (1.15–1.98)
Q4 (>0.171517)	1601/221	2.82 (2.19–3.62)	2.78 (2.16–3.59)	2.60 (2.01–3.36)	1.73 (1.32–2.25)
P for trend	6407/593	<0.001	<0.001	<0.001	<0.001

Model 1 adjusted for age and sex. Model 2 additionally adjusted for education, rural residence, marital status, current smoking, and alcohol use. Model 3 further adjusted for eGFR, LDL-C, ln(C-reactive protein + 0.1), and uric acid and was the primary association model. Model 4 additionally adjusted for baseline CKM stage and was a potentially overadjusted sensitivity model. Q1 was the favorable quartile and Q4 the adverse quartile. Quartiles were defined using sample quantiles calculated with the R type-7 algorithm; eGDR cut points are expressed in mg/kg/min, whereas TyG and AIP are dimensionless. For eGDR, quartiles were ordered from higher to lower values so that higher quartile numbers consistently indicate a more adverse metabolic profile. Trends used the within-quartile median of the original index as a continuous score. AIP, atherogenic index of plasma; CI, confidence interval; CKM, cardiovascular–kidney–metabolic; CMM, cardiometabolic multimorbidity; eGDR, estimated glucose disposal rate; eGFR, estimated glomerular filtration rate; HR, hazard ratio; LDL-C, low-density lipoprotein cholesterol; ln, natural logarithm; SD, standard deviation; TyG, triglyceride–glucose index.

**Figure 2:**
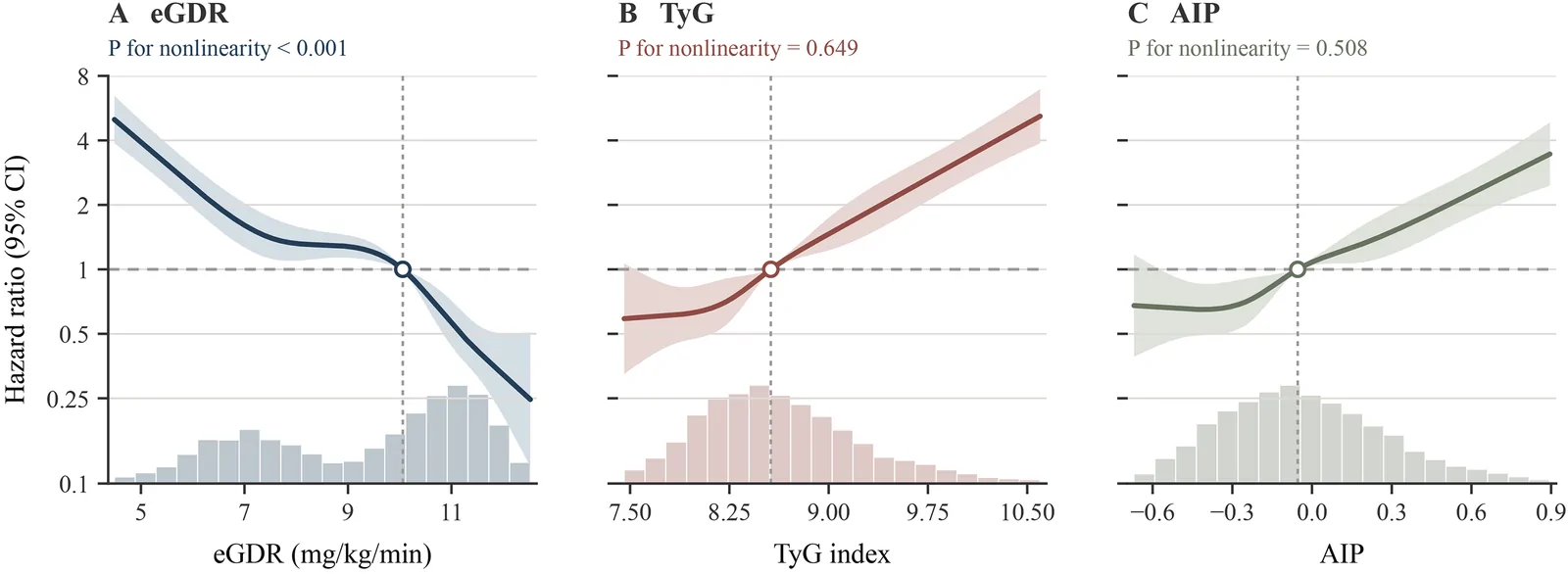
Dose–response associations of eGDR, TyG, and AIP with incident CMM. Restricted cubic-spline associations of eGDR **(A)**, TyG **(B)**, and AIP **(C)** with incident CMM in 6,407 participants with 593 events. Lines and shaded areas represent adjusted HRs and 95% CIs; histograms show index distributions. Five knots were used, with the median as reference. P values were obtained from tests for nonlinearity. AIP, atherogenic index of plasma; CI, confidence interval; CMM, cardiometabolic multimorbidity; eGDR, estimated glucose disposal rate; HR, hazard ratio; TyG, triglyceride–glucose index.

In CKM-stage-adjusted Model 4, HRs were 1.68 (95% CI 1.53–1.85) for lower eGDR and 1.48 (95% CI 1.37–1.61) for higher TyG. The AIP HR was 1.30 (95% CI 1.19–1.42); all P values were <0.001. Global proportional-hazards tests for primary Model 3 showed no nonproportionality for eGDR, TyG, or AIP (P = 0.230, 0.429, and 0.483). The TyG exposure-specific test was borderline (P = 0.064).

### 3.3 Exploratory multistate progression

The multistate cohort included 5,464 participants with no CMM component and 943 with diabetes alone at baseline. During follow-up, 593 developed CMM and 555 died before CMM. Among 6,304 participants with an adjacent determinate interval, 1,382 transitioned from no component to one component and 443 from one component to CMM. Another 112 reached CMM without an observed intervening visit. The preceding state was unknown for 38 CMM events and 52 deaths (**Figure 3**).

**Figure 3:**
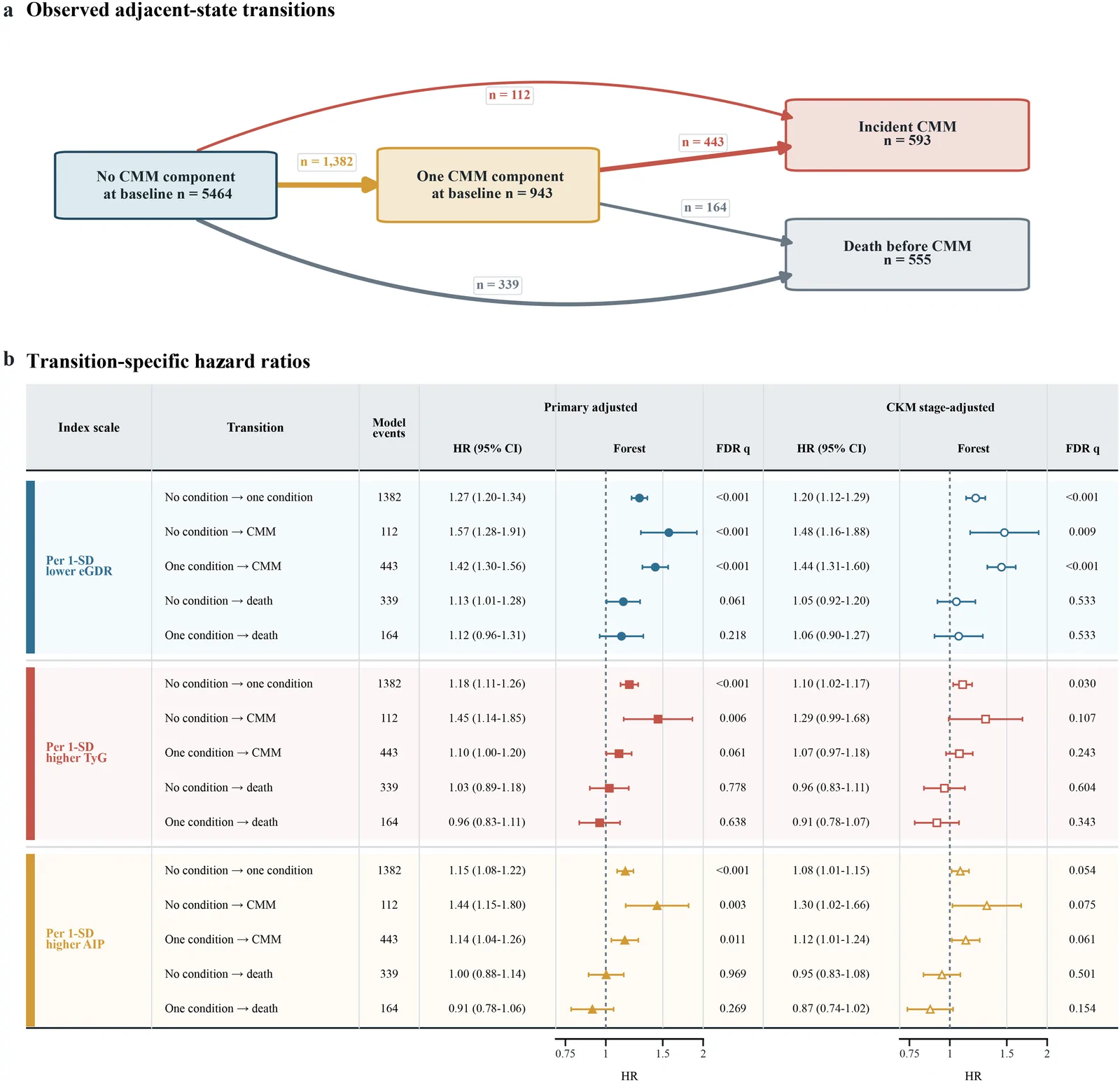
Cardiometabolic progression and transition-specific associations. **(A)** Observed adjacent-state transitions in the complete-case cohort. Arrow widths represent transition counts. All participants with one condition at baseline had diabetes; 38 CMM events and 52 deaths with indeterminate preceding states were not assigned to arrows. **(B)** Transition-specific HRs per 1-SD lower eGDR or higher TyG/AIP. Filled markers show primary adjusted estimates and open markers show estimates additionally adjusted for baseline CKM stage. Horizontal lines denote 95% CIs; q values were adjusted using the Benjamini–Hochberg FDR procedure. AIP, atherogenic index of plasma; CI, confidence interval; CKM, cardiovascular–kidney–metabolic; CMM, cardiometabolic multimorbidity; eGDR, estimated glucose disposal rate; FDR, false discovery rate; HR, hazard ratio; SD, standard deviation; TyG, triglyceride–glucose index.

Model 3 eGDR HRs were 1.27 (95% CI 1.20–1.34), 1.57 (95% CI 1.28–1.91), and 1.42 (95% CI 1.30–1.56), respectively. These estimates corresponded to transitions from no component to one component, no component to CMM, and one component to CMM; all FDR-adjusted P values were <0.001. Corresponding TyG HRs were 1.18 (95% CI 1.11–1.26), 1.45 (95% CI 1.14–1.85), and 1.10 (95% CI 1.00–1.20). FDR-adjusted P values were <0.001, 0.006, and 0.061. Corresponding AIP HRs were 1.15 (95% CI 1.08–1.22), 1.44 (95% CI 1.15–1.80), and 1.14 (95% CI 1.04–1.26). All FDR-adjusted P values were ≤0.011.

After CKM-stage adjustment, eGDR remained associated with all three transitions. The HRs were 1.20 (95% CI 1.12–1.29), 1.48 (95% CI 1.16–1.88), and 1.44 (95% CI 1.31–1.60). All FDR-adjusted P values were ≤0.009. TyG remained associated only with no component to one component (HR 1.10, 95% CI 1.02–1.17; FDR-adjusted P = 0.030), and no AIP estimate survived correction. In disease-specific analyses, eGDR was associated with transitions from no component to diabetes, cardiovascular disease, or CMM and from diabetes to CMM. TyG and AIP were associated with transitions from no component to diabetes and from cardiovascular disease to CMM, but not from diabetes to CMM. No pathway contrast survived correction (minimum FDR-adjusted P = 0.055).

### 3.4 Internal validation of incremental prediction

The base model yielded a C-index of 0.668, 9-year AUC of 0.695, and Brier score of 0.0871 (**Table 4**). Adding eGDR increased the C-index to 0.709 (difference 0.041, 95% CI 0.027–0.055) and AUC to 0.723 (difference 0.028, 95% CI 0.012–0.045). The Brier score improved to 0.0851 (improvement 0.0021, 95% CI 0.0008–0.0032).

**Table 4:** Out-of-fold prediction performance for all eight marker combinations.

Model	C-index	Δ C-index	Holm P	9-year AUC	Δ AUC	Holm P	9-year Brier score	Calibration slope	O/E
Base	0.668	0.000	-	0.695	0.000	-	0.0871	0.910	0.974
+eGDR	0.709	0.041	0.014	0.723	0.028	0.014	0.0851	0.937	0.976
+TyG	0.697	0.029	0.014	0.717	0.022	0.014	0.0857	0.932	0.977
+AIP	0.679	0.011	0.028	0.703	0.008	0.190	0.0867	0.914	0.977
+eGDR+TyG	0.721	0.053	0.014	0.735	0.041	0.014	0.0840	0.944	0.979
+eGDR+AIP	0.713	0.045	0.014	0.728	0.033	0.014	0.0847	0.936	0.978
+TyG+AIP	0.701	0.033	0.014	0.720	0.025	0.014	0.0856	0.936	0.976
+eGDR+TyG+AIP	0.723	0.055	0.014	0.736	0.042	0.014	0.0839	0.945	0.979

The base model included the Model 3 covariates and baseline CKM stage. Performance was estimated using five repetitions of stratified fivefold out-of-fold validation, with preprocessing fitted within each training fold. Lower Brier scores indicate better accuracy. Changes (Δ) are relative to the base model. Holm P values were obtained from 1,000 paired subject-level bootstrap samples and adjusted across the seven model-versus-base comparisons within each metric. AIP, atherogenic index of plasma; AUC, area under the time-dependent receiver operating characteristic curve; C-index, concordance index; eGDR, estimated glucose disposal rate; O/E, observed-to-expected ratio; TyG, triglyceride–glucose index; Δ, change from the base model.

Adding eGDR after TyG improved the C-index by 0.024 (95% CI 0.013–0.034) and AUC by 0.019 (95% CI 0.006–0.031). Reciprocal TyG additions improved these metrics by 0.012 (95% CI 0.005–0.020) and 0.013 (95% CI 0.004–0.022). Adding eGDR after AIP improved the C-index by 0.034 (95% CI 0.021–0.046) and AUC by 0.025 (95% CI 0.010–0.040). Reciprocal AIP additions changed them by 0.004 (95% CI −0.001 to 0.009) and 0.005 (95% CI −0.001 to 0.011).

Adding TyG after AIP improved the C-index by 0.022 (95% CI 0.012–0.033) and AUC by 0.017 (95% CI 0.006–0.030). Reciprocal AIP additions changed them by 0.004 (95% CI −0.001 to 0.010) and 0.003 (95% CI −0.003 to 0.010). Direct eGDR-versus-TyG differences were 0.012 (95% CI −0.004 to 0.027) for the C-index and 0.006 (95% CI −0.014 to 0.024) for AUC. **Table 5** reports all third-marker additions. The eGDR+TyG and three-index models yielded C-indices of 0.721 and 0.723, AUCs of 0.735 and 0.736, and Brier scores of 0.0840 and 0.0839. Adding AIP to eGDR+TyG changed the C-index and AUC by 0.001 each (95% CIs −0.003 to 0.005 and −0.004 to 0.006). Across models, calibration slopes were 0.910–0.945 and observed-to-expected ratios were 0.974–0.979 (**Tables 4**, **5**).

**Table 5:** Paired bootstrap comparisons of prediction performance.

Comparison	Reference	Change in C-index (95% CI)	Holm P	Change in 9-year AUC (95% CI)	Holm P	Brier improvement (95% CI)	Holm P
Single-marker additions to the base model							
eGDR vs Base	Base	0.041 (0.027 to 0.055)	0.006	0.028 (0.012 to 0.045)	0.012	0.0021 (0.0008 to 0.0032)	0.024
TyG vs Base	Base	0.029 (0.016 to 0.043)	0.006	0.022 (0.008 to 0.037)	0.012	0.0014 (0.0002 to 0.0026)	0.056
AIP vs Base	Base	0.011 (0.001 to 0.021)	0.028	0.008 (-0.003 to 0.019)	0.190	0.0005 (-0.0002 to 0.0010)	0.140
Conditional and third-marker additions							
eGDR after TyG	TyG	0.024 (0.013 to 0.034)	0.018	0.019 (0.006 to 0.031)	0.084	0.0017 (0.0005 to 0.0029)	0.112
TyG after eGDR	eGDR	0.012 (0.005 to 0.020)	0.030	0.013 (0.004 to 0.022)	0.084	0.0011 (-0.0001 to 0.0022)	0.384
eGDR after AIP	AIP	0.034 (0.021 to 0.046)	0.018	0.025 (0.010 to 0.040)	0.054	0.0020 (0.0007 to 0.0031)	0.054
AIP after eGDR	eGDR	0.004 (-0.001 to 0.009)	0.402	0.005 (-0.001 to 0.011)	0.342	0.0004 (-0.0002 to 0.0009)	0.919
TyG after AIP	AIP	0.022 (0.012 to 0.033)	0.018	0.017 (0.006 to 0.030)	0.054	0.0011 (-0.0006 to 0.0026)	0.919
AIP after TyG	TyG	0.004 (-0.001 to 0.010)	0.402	0.003 (-0.003 to 0.010)	0.667	0.0002 (-0.0008 to 0.0010)	1.000
eGDR after TyG+AIP	TyG + AIP	0.021 (0.011 to 0.031)	0.018	0.016 (0.003 to 0.028)	0.084	0.0017 (0.0004 to 0.0030)	0.112
TyG after eGDR+AIP	eGDR + AIP	0.010 (0.003 to 0.017)	0.032	0.009 (0.000 to 0.017)	0.176	0.0008 (-0.0006 to 0.0021)	0.919
AIP after eGDR+TyG	eGDR + TyG	0.001 (-0.003 to 0.005)	0.434	0.001 (-0.004 to 0.006)	0.667	0.0001 (-0.0006 to 0.0008)	1.000
Head-to-head single-marker comparisons							
eGDR vs TyG	TyG	0.012 (−0.004 to 0.027)	0.164	0.006 (-0.014 to 0.024)	0.589	0.0006 (-0.0008 to 0.0022)	0.412
eGDR vs AIP	AIP	0.030 (0.014 to 0.044)	0.006	0.020 (0.002 to 0.038)	0.068	0.0016 (0.0003 to 0.0029)	0.090
TyG vs AIP	AIP	0.018 (0.012 to 0.024)	0.006	0.014 (0.008 to 0.022)	0.006	0.0009 (0.0001 to 0.0017)	0.090

Comparisons used 1,000 paired subject-level bootstrap samples. Confidence intervals are unadjusted percentile intervals; displayed P values use Holm correction within each prespecified comparison family and performance metric. Positive Brier improvement denotes a lower score in the expanded or first-named model. AIP, atherogenic index of plasma; AUC, area under the time-dependent receiver operating characteristic curve; CI, confidence interval; C-index, concordance index; eGDR, estimated glucose disposal rate; TyG, triglyceride–glucose index.

### 3.5 Model diagnostics and sensitivity analyses

Discrete-time models included 6,407 participants, 22,383 determinate person-wave intervals, and 593 events. Per adverse 1-SD, Model 3 HRs were 1.92 (95% CI 1.77–2.09) for eGDR and 1.69 (95% CI 1.56–1.83) for TyG. The AIP HR was 1.51 (95% CI 1.39–1.65) (**Supplementary Material 2**; **Supplementary Table S1**). In the analysis restricted to outcome ascertainment through 2018 (6,385 participants and 420 events), associations for all three indices were similar in direction and magnitude to the primary results (**Supplementary Material 2**; **Supplementary Table S4**).

Cox models excluding participants with baseline diabetes included 5,464 participants and 328 CMM events. Associations for all three indices remained significant in Models 3 and 4, with no evidence of nonproportionality from exposure-specific or global Schoenfeld tests (**Supplementary Material 2**; **Supplementary Table S2**).

With death as a competing event, 593 CMM events and 555 deaths were observed. Per adverse 1-SD, cause-specific HRs were 1.92 (95% CI 1.77–2.08) for eGDR and 1.70 (95% CI 1.58–1.82) for TyG. The AIP HR was 1.52 (95% CI 1.40–1.65). Corresponding Fine–Gray sHRs were 1.90 (95% CI 1.75–2.07), 1.69 (95% CI 1.57–1.83), and 1.52 (95% CI 1.39–1.66). Aalen-Johansen cumulative incidence increased across adverse quartiles (Gray’s test, all P < 0.001). All Model 3 associations remained significant after Holm and Benjamini–Hochberg correction (**Supplementary Material 2**; **Supplementary Table S3**; **Supplementary Figure S1**).

Scaled Schoenfeld diagnostics showed no nonproportionality in Model 3 baseline-diabetes-free or cause-specific Cox models. In cause-specific Model 4, global tests were significant (P = 0.010–0.017), and the TyG exposure-specific test gave P = 0.044.

Bridge-state and piecewise exponential estimates resembled those from adjacent-interval models, and no time-by-index test was significant. No death transition met the FDR threshold. The direct no-component-to-CMM transition had 112 events, corresponding to 7.0 events per parameter in Model 3 and 5.9 in Model 4. These estimates remained exploratory.

## 4 Discussion

Among middle-aged and older Chinese adults with CKM stages 0–3 and no CMM at baseline, all three indices were associated with incident CMM over 9 years of follow-up. The associations persisted in analyses addressing interval-censored event timing, baseline diabetes, competing death, and restriction of outcome ascertainment through 2018. eGDR showed the broadest transition associations and the numerically largest single-marker gain in internally validated prediction. TyG added complementary information, whereas AIP contributed little after eGDR and TyG were included.

These findings are consistent with previous CHARLS studies linking TyG-related indices to CMM (8–10) and with recent comparisons in which eGDR showed the highest discrimination among insulin-resistance surrogates (9–12). The present analysis extends that evidence by evaluating overall CMM incidence, transition-specific disease accumulation, and all eight marker combinations. This design directly addresses whether TyG or AIP contributes information beyond eGDR, rather than comparing single-marker performance alone.

The broader associations observed for eGDR may partly reflect its greater overlap with established CKM risk domains. Although all three indices share information with CKM staging, eGDR incorporates the widest range of stage-defining features. Waist circumference contributes to adiposity and metabolic syndrome criteria, hypertension contributes to stage 2 classification, and HbA1c informs prediabetes and diabetes status. HbA1c also contributes to baseline diabetes ascertainment, and diabetes is one of the three components of CMM, whereas waist circumference and hypertension do not directly define CMM. This broader cardiometabolic content may contribute to the stronger associations and the numerically largest single-marker improvement observed for eGDR, in addition to its role as a surrogate measure of insulin resistance. Adjustment for CKM stage attenuated the eGDR association, whereas the association remained evident after excluding participants with baseline diabetes, indicating that it was not explained solely by baseline diabetes status. Because CKM staging incorporates several components of the indices, such adjustment may also remove part of the prognostic information captured by eGDR. Taken together, eGDR may function as an integrative marker of insulin resistance and broader cardiometabolic risk, with its broader associations and numerically larger single-marker gain likely reflecting both dimensions rather than a purely insulin-resistance-specific advantage.

Restricted cubic spline analyses showed a nonlinear inverse association between eGDR and CMM risk, whereas TyG and AIP showed broadly linear positive associations. The eGDR curve was steepest at lower values and flatter across much of the upper distribution. Confidence intervals widened at both extremes, and no threshold was formally estimated; consequently, clinically meaningful cutoffs cannot be inferred from these curves.

In the multistate analysis, eGDR remained associated with all three disease-state transitions after adjustment for CKM stage. The association for TyG remained significant after FDR correction only for the transition from no cardiometabolic disease component to one component, whereas none of the associations between AIP and the individual transitions remained significant. Disease-specific analyses further indicated that eGDR was associated both with the onset of a first cardiometabolic condition and with progression from diabetes to CMM. These findings should nevertheless be considered exploratory because disease onset was interval-censored, the preceding disease state could not be established for some participants, and relatively few events informed each parameter in the direct-transition model. A direct transition from no cardiometabolic disease components to CMM may reflect an unobserved intermediate state arising between study visits rather than the simultaneous onset of two conditions.

Adding eGDR to the base prediction model increased the C-index by 0.041 and the 9-year AUC by 0.028, while improving the Brier score by 0.0021. By comparison, adding AIP to a model already containing eGDR and TyG increased both the C-index and AUC by only 0.001. The strong correlation between TyG and AIP likely reflects their shared triglyceride component and may partly explain the limited incremental value of AIP in models already containing TyG. Calibration was generally close to ideal, although the absolute improvement in the Brier score was modest. The direct eGDR-versus-TyG difference in C-index was modest, and its 95% CI included zero (difference, 0.012; 95% CI, −0.004 to 0.027). Although these indices may complement CKM staging and PREVENT-based risk assessment (2, 18), external validation, assessment of net benefit, and clearly prespecified risk thresholds are needed before they can be used clinically (22, 28).

The strengths of this study include the 9-year follow-up, direct comparison of three routinely available indices within a common primary cohort, multistate models that treated death as a competing event, and repeated out-of-fold internal validation of predictive performance. Several limitations should be considered. First, the observational design precludes causal inference, and residual confounding cannot be excluded. Second, heart disease and stroke were primarily self-reported, which may have introduced outcome misclassification. Comparable medication data were unavailable in 2020, but estimates were similar when outcome ascertainment was restricted through 2018. Third, event times were approximated using the midpoint between consecutive survey visits. Fourth, indices measured only at baseline could not capture changes in metabolic status or treatment. Fifth, CKM staging was adapted to variables available in CHARLS. Sixth, prediction performance was internally validated only. Seventh, although observed baseline differences between included and excluded participants were generally modest, selection bias cannot be excluded. Finally, because eGDR has broader component overlap with CKM staging and baseline diabetes ascertainment than the other indices, its stronger associations and numerically larger predictive gain may partly reflect embedded cardiometabolic risk information.

Future studies should externally validate these findings in populations from different countries, racial and ethnic groups, and levels of cardiometabolic risk to assess the generalizability and incremental predictive value of eGDR, TyG, and AIP. Further work is also needed to investigate the mechanisms linking insulin resistance to CMM progression. Prospective validation should establish clinically meaningful risk thresholds and determine whether combining eGDR and TyG provides sufficient incremental value for identifying high-risk individuals and supporting clinical decisions.

### 4.1 Conclusion

Among Chinese adults with CKM stages 0–3, lower eGDR, higher TyG, and higher AIP were associated with incident CMM. eGDR showed the broadest transition pattern and the numerically largest single-marker gain in internally validated prediction; TyG added complementary information, whereas AIP added little beyond eGDR and TyG. These findings support further evaluation of eGDR and TyG for early CKM risk stratification but require external validation and assessment of clinical net benefit.

## Data Availability

The datasets analyzed during the current study are available from the China Health and Retirement Longitudinal Study (CHARLS) upon registration and approval of a data-use application at https://charls.pku.edu.cn/. The analytic code used in this study is available from the corresponding author on reasonable request.

## References

[1] Ndumele CE Rangaswami J Chow SL Neeland IJ Tuttle KR Khan SS . Cardiovascular-kidney-metabolic health: a presidential advisory from the American Heart Association. Circulation. (2023) 148:1606–35. doi: 10.1161/CIR.0000000000001184 37807924

[2] Ndumele CE Rodriguez F Dixon DL Khan SS Mukherjee D Writing Committee Members . 2026 AHA/ACC/ADA/ASN guideline for the prevention, detection, evaluation, and management of cardiovascular-kidney-metabolic syndrome: a report of the American College of Cardiology/American Heart Association Joint Committee on Clinical Practice Guidelines. Circulation. (2026) 154:e50–e158. doi: 10.1161/CIR.0000000000001453 42263157

[3] Emerging Risk Factors Collaboration Di Angelantonio E Kaptoge S Wormser D Willeit P Butterworth AS . Association of cardiometabolic multimorbidity with mortality. JAMA. (2015) 314:52–60. doi: 10.1001/jama.2015.7008 26151266 PMC4664176

[4] Skou ST Mair FS Fortin M Guthrie B Nunes BP Miranda JJ . Multimorbidity. Nat Rev Dis Primers. (2022) 8:48. doi: 10.1038/s41572-022-00376-4 35835758 PMC7613517

[5] Williams KV Erbey JR Becker D Arslanian S Orchard TJ . Can clinical factors estimate insulin resistance in type 1 diabetes? Diabetes. (2000) 49:626–32. doi: 10.2337/diabetes.49.4.626 10871201

[6] Guerrero-Romero F Simental-Mendía LE González-Ortiz M Martínez-Abundis E Ramos-Zavala MG Hernández-González SO . The product of triglycerides and glucose, a simple measure of insulin sensitivity. Comparison with the euglycemic-hyperinsulinemic clamp. J Clin Endocrinol Metab. (2010) 95:3347–51. doi: 10.1210/jc.2010-0288 20484475

[7] Dobiášová M Frohlich J . The plasma parameter log (TG/HDL-C) as an atherogenic index: correlation with lipoprotein particle size and esterification rate in apoB-lipoprotein-depleted plasma (FER(HDL)). Clin Biochem. (2001) 34:583–8. doi: 10.1016/S0009-9120(01)00263-6 11738396

[8] Zhang Z Zhao L Lu Y Meng X Zhou X . Relationship of triglyceride-glucose index with cardiometabolic multi-morbidity in China: evidence from a national survey. Diabetol Metab Syndr. (2023) 15:226. doi: 10.1186/s13098-023-01205-8 37926824 PMC10626797

[9] Lai J Liu Z Wang H Kong X Wei Y Cao Y . Assessment of seven insulin resistance surrogate indexes for predicting cardiometabolic multimorbidity among Chinese middle-aged and older adults: a national prospective cohort study. Cardiovasc Diabetol. (2026) 25:121. doi: 10.1186/s12933-026-03168-2 41937148 PMC13067570

[10] Zhao E Xie H Wang R Dong B Zhang C . Estimated glucose disposal rate outperforms thirteen other insulin resistance indices in predicting new-onset cardiometabolic multimorbidity: a longitudinal study from the China Health and Retirement Longitudinal Study (CHARLS) and cross-sectional replication from the National Health and Nutrition Examination Survey (NHANES). Cardiovasc Diabetol. (2026) 25:205. doi: 10.1186/s12933-026-03218-9 42226282 PMC13390194

[11] Dong B Chen Y Yang X Chen Z Zhang H Gao Y . Estimated glucose disposal rate outperforms other insulin resistance surrogates in predicting incident cardiovascular diseases in cardiovascular-kidney-metabolic syndrome stages 0-3 and the development of a machine learning prediction model: a nationwide prospective cohort study. Cardiovasc Diabetol. (2025) 24:163. doi: 10.1186/s12933-025-02729-1 40241176 PMC12004813

[12] Xue N Ma L Zhou Z Bai T Jia X Wei X . Insulin resistance surrogate indices and incident cardiovascular disease across cardiovascular-kidney-metabolic stages 0-3: a prospective cohort study. Cardiovasc Diabetol. (2026) 25:226. doi: 10.1186/s12933-026-03280-3 42380905 PMC13465377

[13] Liu Z Zhou D Tang Y Huo G . Association of atherogenic index of plasma with stroke risk in a population with cardiovascular-kidney-metabolic syndrome stages 0 to 3: the first evidence from the China Health and Retirement Longitudinal Study. J Am Heart Assoc. (2025) 14:e039983. doi: 10.1161/JAHA.124.039983 40654258 PMC12533614

[14] Wu H Zhou Y Lai X Ma B Ding L Zhu X . Association between metabolic score for insulin resistance and future stroke risk in patients with cardiovascular-kidney-metabolic syndrome stages 0-3: a longitudinal analysis based on CHARLS. Cardiovasc Diabetol. (2025) 24:379. doi: 10.1186/s12933-025-02932-0 41035026 PMC12486780

[15] Zhou X Liu Y Peng J Zhou X Wei H . The role of triglyceride-glucose index in the progression of cardiovascular-kidney-metabolic syndrome: findings from the China Health and Retirement Longitudinal Study. Front Cardiovasc Med. (2025) 12:1612121. doi: 10.3389/fcvm.2025.1612121 40625397 PMC12230094

[16] Qian J Chi Q Qian C Fan X Ding W Wang T . Atherogenic index of plasma and triglyceride-glucose index mediate the association between stroke and all-cause mortality: insights from the lipid paradox. Lipids Health Dis. (2025) 24:173. doi: 10.1186/s12944-025-02586-7 40349063 PMC12065248

[17] Zhao Y Hu Y Smith JP Strauss J Yang G . Cohort profile: the China Health and Retirement Longitudinal Study (CHARLS). Int J Epidemiol. (2014) 43:61–8. doi: 10.1093/ije/dys203 23243115 PMC3937970

[18] Khan SS Matsushita K Sang Y Ballew SH Grams ME Surapaneni A . Development and validation of the American Heart Association’s PREVENT equations. Circulation. (2024) 149:430–49. doi: 10.1161/CIRCULATIONAHA.123.067626 37947085 PMC10910659

[19] Inker LA Eneanya ND Coresh J Tighiouart H Wang D Sang Y . New creatinine- and cystatin C-based equations to estimate GFR without race. N Engl J Med. (2021) 385:1737–49. doi: 10.1056/NEJMoa2102953 34554658 PMC8822996

[20] Austin PC Lee DS Fine JP . Introduction to the analysis of survival data in the presence of competing risks. Circulation. (2016) 133:601–9. doi: 10.1161/CIRCULATIONAHA.115.017719 26858290 PMC4741409

[21] Putter H Fiocco M Geskus RB . Tutorial in biostatistics: competing risks and multi-state models. Stat Med. (2007) 26:2389–430. doi: 10.1002/sim.2712 17031868

[22] Collins GS Dhiman P Ma J Schlussel MM Archer L Van Calster B . Evaluation of clinical prediction models (part 1): from development to external validation. BMJ. (2024) 384:e074819. doi: 10.1136/bmj-2023-074819 38191193 PMC10772854

[23] Steyerberg EW Vickers AJ Cook NR Gerds T Gonen M Obuchowski N . Assessing the performance of prediction models: a framework for traditional and novel measures. Epidemiology. (2010) 21:128–38. doi: 10.1097/EDE.0b013e3181c30fb2 20010215 PMC3575184

[24] Uno H Cai T Pencina MJ D’Agostino RB Wei LJ . On the C-statistics for evaluating overall adequacy of risk prediction procedures with censored survival data. Stat Med. (2011) 30:1105–17. doi: 10.1002/sim.4154 21484848 PMC3079915

[25] Heagerty PJ Lumley T Pepe MS . Time-dependent ROC curves for censored survival data and a diagnostic marker. Biometrics. (2000) 56:337–44. doi: 10.1111/j.0006-341X.2000.00337.x 10877287

[26] Gerds TA Schumacher M . Consistent estimation of the expected Brier score in general survival models with right-censored event times. Biom J. (2006) 48:1029–40. doi: 10.1002/bimj.200610301 17240660

[27] Van Calster B McLernon DJ van Smeden M Wynants L Steyerberg EW Topic Group ‘Evaluating Diagnostic Tests and Prediction Models’ of the STRATOS Initiative . Calibration: the Achilles heel of predictive analytics. BMC Med. (2019) 17:230. doi: 10.1186/s12916-019-1466-7 31842878 PMC6912996

[28] Vickers AJ Elkin EB . Decision curve analysis: a novel method for evaluating prediction models. Med Decis Making. (2006) 26:565–74. doi: 10.1177/0272989X06295361 17099194 PMC2577036

