# Supplementary material for: Estimated glucose disposal rate shows the broadest associations with cardiometabolic multimorbidity progression in early cardiovascular–kidney–metabolic stages: a longitudinal cohort study
Source: Front Endocrinol (Lausanne). 2026 Sep 30;17:1948249. doi: 10.3389/fendo.2026.1948249 (PMC13662789; doi:10.3389/fendo.2026.1948249)
Supplement: Supplementary file 1 [file Supplementaryfile1.docx]

Supplementary Material 1

Estimated glucose disposal rate shows the broadest associations with cardiometabolic multimorbidity progression in early cardiovascular–kidney–metabolic stages: a longitudinal cohort study

# 1 Supplementary methods

## 1.1 Cohort construction and exposure assessment

Eligibility was assessed using data from the 2011 CHARLS survey. Participants were aged 45–79 years, had fasted before blood collection, and had valid values for all three indices. They also required determinate absence of heart disease and stroke at baseline, at least one determinate follow-up CMM assessment, and a classifiable baseline CKM stage of 0–3. After this stage-determinate cohort was defined, 9 of 6,416 participants (0.14%) were excluded for missing LDL-C, leaving a final complete-case cohort of 6,407. All other required baseline exposures and model covariates were complete; no baseline values were imputed. Within the age-eligible, baseline-CVD-free source population, baseline characteristics of included and excluded participants were compared using standardized mean differences (SMDs) (Supplementary Material 2: Table S5). No formal sample-size calculation was performed; all participants meeting the prespecified eligibility criteria were included.

Special codes, nonnumeric entries, and nonfinite values were treated as missing. Waist circumference values outside 50–160 cm were treated as missing, and fasting glucose, triglycerides, HDL cholesterol, and HbA1c had to be positive and finite. eGDR was calculated as 21.158 − 0.09 × waist circumference (cm) − 3.407 × hypertension − 0.551 × HbA1c (%), with its hypertension component defined as systolic blood pressure ≥140 mmHg or diastolic blood pressure ≥90 mmHg, or documented diagnosis or antihypertensive treatment. TyG was ln[triglycerides (mg/dL) × fasting glucose (mg/dL)/2], and AIP was log10[triglycerides (mmol/L)/HDL cholesterol (mmol/L)]. eGDR was multiplied by −1 before standardization so that higher standardized values represented a more adverse metabolic profile, and standardized in the stage-determinate source cohort before complete-case restriction so that higher values indicated greater adverse exposure.

## 1.2 CKM-stage operationalization

Baseline CKM stage was assigned from high to low using a CHARLS-adapted implementation of the 2026 AHA/ACC/ADA/ASN guideline. Stage 1 included BMI at least 23 kg/m², central obesity (waist at least 90 cm in men or 80 cm in women), or prediabetes. Stage 2 included hypertension, diabetes, triglycerides at least 150 mg/dL, metabolic syndrome, or chronic kidney disease. Chronic kidney disease was defined by eGFR below 60 mL/min/1.73 m² or a self-reported physician-diagnosed kidney disease. For CKM staging, hypertension was defined separately as systolic blood pressure ≥130 mmHg or diastolic blood pressure ≥80 mmHg, or documented diagnosis or antihypertensive treatment. The ≥140/90 mmHg threshold was used only for the hypertension component of the established eGDR equation, whereas the ≥130/80 mmHg threshold was used for CKM-stage assignment.

Stage 3 was defined by eGFR below 30 mL/min/1.73 m² or a 10-year total cardiovascular disease risk of at least 20% from the base PREVENT equation. eGFR used the 2021 race-free CKD-EPI creatinine equation, and PREVENT inputs outside accepted ranges were set to the nearest allowed value. Baseline heart disease or stroke was classified as stage 4 and excluded. Stage 0 required all available and observed stage 1–3 criteria to be negative. Missing values for available criteria were not interpreted as absence; unavailable criteria were not incorporated into staging. Urine albumin-to-creatinine ratio and direct measures of subclinical atherosclerotic disease or pre-heart failure were unavailable and were treated as unmeasured.

## 1.3 CMM ascertainment and follow-up

Diabetes, heart disease, and stroke were treated as cumulative conditions. During 2011–2018, a condition was considered present based on a self-reported physician diagnosis or corresponding medication use. Baseline diabetes also included fasting glucose of at least 126 mg/dL or HbA1c of at least 6.5%. An explicit report of no diagnosis, without positive medication evidence, indicated absence; unresolved evidence was coded as unknown. Previously confirmed disease was carried forward. Because equivalent medication items were unavailable in 2020, newly identified disease at that wave relied on self-reported physician diagnoses alone.

CMM was defined as the coexistence of at least two of the three conditions. The primary event time was the midpoint between the last determinate CMM-negative survey and the first positive survey. Participants without CMM were censored at their last determinate negative survey. A discrete-time analysis was used to assess sensitivity to this event-time approximation.

## 1.4 Association and dose–response analyses

Continuous variables were summarized as median (interquartile range) and categorical variables as n (%). Baseline covariates were selected a priori: age, sex, education, rural residence, marital status, current smoking, alcohol use in the previous year, eGFR, LDL cholesterol, ln(C-reactive protein + 0.1), and uric acid. Model 1 included age and sex; Model 2 added socioeconomic and behavioral factors; the primary Model 3 additionally included the laboratory covariates. Model 4 added CKM stage and was considered a sensitivity model because stage overlaps metabolically with the indices. Components used to calculate an index were not entered separately.

Cox models used time since the 2011 interview and reported hazard ratios with 95% CIs per 1-SD adverse difference. Proportional hazards were assessed with Schoenfeld residuals. Each index was also examined in adverse-direction quartiles, with Q4 denoting the lowest eGDR or highest TyG or AIP; trend tests used the within-quartile median. Restricted cubic splines used five knots at the 5th, 27.5th, 50th, 72.5th, and 95th percentiles, with the median as reference. Curves were displayed between the 1st and 99th percentiles, and nonlinearity was assessed by comparing spline and linear models. Primary models were unweighted because the estimand was the conditional association within the complete-case analytic cohort rather than a population-representative association.

## 1.5 Exploratory multistate analysis

The multistate analysis used the same 6,407 participants. At each wave, cumulative disease evidence classified participants as having no CMM component, one component, CMM, or an unknown state; death before CMM was absorbing. Primary analyses used adjacent intervals with determinate origin and destination states. Modeled transitions were no component to one component, no component to CMM without an observed intermediate visit, one component to CMM, and transitions from the first two states to death. The direct transition to CMM may reflect an unobserved intermediate state and was interpreted accordingly.

Transition-specific complementary log-log models included survey-interval indicators and participant-clustered robust standard errors. Each index was entered separately with Model 3 covariates; Model 4 added CKM stage. Benjamini–Hochberg correction covered the 15 index-transition tests within each model. Secondary analyses bridged missing waves, used piecewise exponential models, separated diabetes-only from cardiovascular-disease-only states, and tested variation across intervals and pathways. All multistate analyses were exploratory.

## 1.6 Internal validation of incremental prediction

Nine-year CMM prediction used the complete-case cohort and a base model containing the Model 3 covariates plus CKM stage. Eight prespecified models represented every combination of eGDR, TyG, and AIP. Performance was estimated using five repeats of stratified fivefold out-of-fold validation, with identical folds across models. Within each cross-validation split, standardization parameters were estimated exclusively from the four training folds and applied unchanged to the held-out fold. Participant-level predictions were averaged across repeats.

Performance measures were the C-index, 9-year cumulative/dynamic AUC, inverse-probability-of-censoring weighted Brier score, calibration slope, observed-to-expected ratio, and calibration plots. Paired participant bootstrap resampling with 1,000 replicates provided 95% CIs for 15 prespecified comparisons: single-index additions to the base model, reciprocal second-index additions, third-index additions, and direct comparisons among the three single-index models. Holm and Benjamini–Hochberg adjusted P values were reported within comparison families.

## 1.7 Sensitivity analyses

Sensitivity analyses were derived from the fixed complete-case cohort. The discrete-time and competing-risk analyses retained all 6,407 participants, whereas the baseline-diabetes-free Cox analysis included 5,464 participants. Interval-censored outcome ascertainment was represented as determinate person-wave records and analyzed using complementary log-log models with survey-interval indicators and participant-clustered robust standard errors. Cox models were also repeated after excluding participants with baseline diabetes. Death before CMM was treated as a competing event using cause-specific Cox and Fine–Gray models; Aalen–Johansen estimates described cumulative incidence.

To assess the effect of non-comparable medication ascertainment in 2020, we repeated Cox models with follow-up restricted through 2018. The 2011 time origin, baseline exposures and covariates, model specifications, and Efron handling of ties were unchanged. Outcome ascertainment used only the 2013, 2015, and 2018 follow-up surveys. Event times retained the primary midpoint definition, whereas non-events were censored at their last determinate CMM-negative survey through 2018. Participants without a determinate post-baseline CMM status through 2018 were excluded only from this sensitivity analysis. After excluding 22 such participants, the analysis included 6,385 participants and 420 CMM events.

Model 3 was primary, and Model 4 additionally included baseline CKM stage. Scaled Schoenfeld residuals assessed proportional hazards. Holm and Benjamini–Hochberg corrections covered the three indices within each model and method. Tests were two-sided, with P < 0.05 used as the nominal threshold. Analyses used R 4.5.2 and Python 3.14.3.

# 2 Abbreviations

AIP, atherogenic index of plasma; BMI, body mass index; CHARLS, China Health and Retirement Longitudinal Study; CKM, cardiovascular–kidney–metabolic; CMM, cardiometabolic multimorbidity; eGDR, estimated glucose disposal rate; eGFR, estimated glomerular filtration rate; HbA1c, glycated hemoglobin; HDL, high-density lipoprotein; LDL, low-density lipoprotein; PREVENT, Predicting Risk of Cardiovascular Disease EVENTs; TyG, triglyceride–glucose index.
