# Supplementary material for: Estimated glucose disposal rate shows the broadest associations with cardiometabolic multimorbidity progression in early cardiovascular–kidney–metabolic stages: a longitudinal cohort study
Source: Front Endocrinol (Lausanne). 2026 Sep 30;17:1948249. doi: 10.3389/fendo.2026.1948249 (PMC13662789; doi:10.3389/fendo.2026.1948249)
Supplement: Supplementary file 2 [file Supplementaryfile2.docx]

Supplementary Material 2

Estimated glucose disposal rate shows the broadest associations with cardiometabolic multimorbidity progression in early cardiovascular–kidney–metabolic stages: a longitudinal cohort study

# 1 Supplementary tables and figures

Table S1. Interval-aware discrete-time complementary log–log models

| **Model** | **Index and contrast** | **Participants** | **Person-wave intervals** | **CMM events** | **HR (95% CI)** | **P value** | **Holm-adjusted P** | **BH-adjusted P** |
| --- | --- | --- | --- | --- | --- | --- | --- | --- |
| Model 3 | eGDR (per 1-SD lower) | 6,407 | 22,383 | 593 | 1.92 (1.77–2.09) | <0.001 | <0.001 | <0.001 |
| Model 3 | TyG (per 1-SD higher) | 6,407 | 22,383 | 593 | 1.69 (1.56–1.83) | <0.001 | <0.001 | <0.001 |
| Model 3 | AIP (per 1-SD higher) | 6,407 | 22,383 | 593 | 1.51 (1.39–1.65) | <0.001 | <0.001 | <0.001 |
| Model 4 | eGDR (per 1-SD lower) | 6,407 | 22,383 | 593 | 1.68 (1.52–1.86) | <0.001 | <0.001 | <0.001 |
| Model 4 | TyG (per 1-SD higher) | 6,407 | 22,383 | 593 | 1.48 (1.36–1.61) | <0.001 | <0.001 | <0.001 |
| Model 4 | AIP (per 1-SD higher) | 6,407 | 22,383 | 593 | 1.30 (1.19–1.43) | <0.001 | <0.001 | <0.001 |

*Note.* Determinate person-wave records were analyzed with survey-interval indicators and participant-clustered robust standard errors. The risk set contained 6,407 participants, 22,383 intervals, and 593 events; 2,618 unknown person-waves were excluded. Model 3 was primary; Model 4 additionally adjusted for baseline CKM stage. Holm and Benjamini–Hochberg (BH) corrections covered the three indices within each model. Abbreviations: AIP, atherogenic index of plasma; BH, Benjamini–Hochberg; CI, confidence interval; CKM, cardiovascular–kidney–metabolic; CMM, cardiometabolic multimorbidity; eGDR, estimated glucose disposal rate; HR, hazard ratio; SD, standard deviation; TyG, triglyceride–glucose index.

Table S2. Cox models excluding participants with baseline diabetes

| **Model** | **Index and contrast** | **Participants** | **CMM events** | **HR (95% CI)** | **P value** | **PH P (index)** | **PH P (global)** | **Holm-adjusted P** | **BH-adjusted P** |
| --- | --- | --- | --- | --- | --- | --- | --- | --- | --- |
| Model 3 | eGDR (per 1-SD lower) | 5,464 | 328 | 1.60 (1.42–1.79) | <0.001 | 0.397 | 0.804 | <0.001 | <0.001 |
| Model 3 | TyG (per 1-SD higher) | 5,464 | 328 | 1.39 (1.22–1.59) | <0.001 | 0.579 | 0.735 | <0.001 | <0.001 |
| Model 3 | AIP (per 1-SD higher) | 5,464 | 328 | 1.36 (1.20–1.53) | <0.001 | 0.544 | 0.743 | <0.001 | <0.001 |
| Model 4 | eGDR (per 1-SD lower) | 5,464 | 328 | 1.46 (1.28–1.67) | <0.001 | 0.363 | 0.507 | <0.001 | <0.001 |
| Model 4 | TyG (per 1-SD higher) | 5,464 | 328 | 1.21 (1.05–1.40) | 0.007 | 0.612 | 0.361 | 0.009 | 0.007 |
| Model 4 | AIP (per 1-SD higher) | 5,464 | 328 | 1.21 (1.06–1.37) | 0.004 | 0.578 | 0.354 | 0.009 | 0.007 |

*Note.* The analysis included 5,464 participants and 328 incident CMM events. Model 3 was primary; Model 4 additionally adjusted for baseline CKM stage. PH P values are based on scaled Schoenfeld residuals and showed no evidence of nonproportionality. Holm and BH corrections covered the three indices within each model. Abbreviations: AIP, atherogenic index of plasma; BH, Benjamini–Hochberg; CI, confidence interval; CKM, cardiovascular–kidney–metabolic; CMM, cardiometabolic multimorbidity; eGDR, estimated glucose disposal rate; HR, hazard ratio; PH, proportional hazards; SD, standard deviation; TyG, triglyceride–glucose index.

Table S3, Panel A. Competing-risk analysis treating death before CMM as a competing event: cause-specific Cox and Fine–Gray estimates

| **Model** | **Index and contrast** | **Cause-specific HR (95% CI)** | **P value** | **Fine–Gray sHR (95% CI)** | **P value** | **Holm P (CS / FG)** | **BH P (CS / FG)** |
| --- | --- | --- | --- | --- | --- | --- | --- |
| Model 3 | eGDR (per 1-SD lower) | 1.92 (1.77–2.08) | <0.001 | 1.90 (1.75–2.07) | <0.001 | <0.001 / <0.001 | <0.001 / <0.001 |
| Model 3 | TyG (per 1-SD higher) | 1.70 (1.58–1.82) | <0.001 | 1.69 (1.57–1.83) | <0.001 | <0.001 / <0.001 | <0.001 / <0.001 |
| Model 3 | AIP (per 1-SD higher) | 1.52 (1.40–1.65) | <0.001 | 1.52 (1.39–1.66) | <0.001 | <0.001 / <0.001 | <0.001 / <0.001 |
| Model 4 | eGDR (per 1-SD lower) | 1.68 (1.53–1.85) | <0.001 | 1.68 (1.52–1.85) | <0.001 | <0.001 / <0.001 | <0.001 / <0.001 |
| Model 4 | TyG (per 1-SD higher) | 1.49 (1.38–1.62) | <0.001 | 1.50 (1.38–1.63) | <0.001 | <0.001 / <0.001 | <0.001 / <0.001 |
| Model 4 | AIP (per 1-SD higher) | 1.31 (1.20–1.43) | <0.001 | 1.32 (1.20–1.45) | <0.001 | <0.001 / <0.001 | <0.001 / <0.001 |

*Note.* The analysis included 6,407 participants, 593 CMM events, 555 competing deaths, and 5,259 event-free censorings. Model 3 was primary; Model 4 additionally adjusted for baseline CKM stage. Cause-specific Model 3 showed no evidence of nonproportionality. In Model 4, global PH tests were significant (P = 0.010–0.017), and the TyG exposure-specific test gave P = 0.044. Holm and BH corrections covered the three indices within each model and method. Abbreviations: AIP, atherogenic index of plasma; BH, Benjamini–Hochberg; CI, confidence interval; CKM, cardiovascular–kidney–metabolic; CMM, cardiometabolic multimorbidity; CS, cause-specific; eGDR, estimated glucose disposal rate; FG, Fine–Gray; HR, hazard ratio; PH, proportional hazards; SD, standard deviation; sHR, subdistribution hazard ratio; TyG, triglyceride–glucose index.

Table S3, Panel B. Aalen–Johansen 9-year cumulative incidence by adverse-direction quartile

| **Index** | **Quartile** | **Participants** | **CMM events** | **Competing deaths** | **9-year CMM CIF, % (95% CI)** | **9-year death CIF, % (95% CI)** | **Gray P (CMM)** |
| --- | --- | --- | --- | --- | --- | --- | --- |
| eGDR | Q1 | 1,603 | 47 | 131 | 3.0 (2.2–3.8) | 8.1 (6.7–9.6) | <0.001 |
| eGDR | Q2 | 1,601 | 94 | 93 | 6.1 (4.9–7.3) | 5.4 (4.2–6.6) | <0.001 |
| eGDR | Q3 | 1,601 | 156 | 170 | 10.1 (8.6–11.6) | 10.7 (9.1–12.3) | <0.001 |
| eGDR | Q4 | 1,602 | 296 | 161 | 19.1 (17.1–21.0) | 9.9 (8.3–11.4) | <0.001 |
| TyG | Q1 | 1,602 | 72 | 154 | 4.6 (3.6–5.7) | 9.4 (7.9–10.9) | <0.001 |
| TyG | Q2 | 1,602 | 109 | 127 | 7.0 (5.7–8.2) | 7.8 (6.4–9.1) | <0.001 |
| TyG | Q3 | 1,601 | 150 | 152 | 9.7 (8.2–11.1) | 9.0 (7.5–10.5) | <0.001 |
| TyG | Q4 | 1,602 | 262 | 122 | 17.0 (15.1–18.9) | 7.9 (6.5–9.4) | <0.001 |
| AIP | Q1 | 1,603 | 84 | 158 | 5.4 (4.2–6.5) | 9.6 (8.1–11.1) | <0.001 |
| AIP | Q2 | 1,602 | 119 | 146 | 7.6 (6.3–9.0) | 8.7 (7.3–10.2) | <0.001 |
| AIP | Q3 | 1,601 | 169 | 138 | 10.9 (9.4–12.5) | 8.5 (7.1–9.9) | <0.001 |
| AIP | Q4 | 1,601 | 221 | 113 | 14.3 (12.5–16.0) | 7.2 (5.9–8.6) | <0.001 |

*Note.* Gray tests compared CMM cumulative incidence across quartiles within each index. Quartiles are oriented from favorable (Q1) to adverse (Q4); Q4 is the lowest eGDR or the highest TyG or AIP. Values are Aalen–Johansen estimates with pointwise 95% CIs. Abbreviations: AIP, atherogenic index of plasma; CI, confidence interval; CIF, cumulative incidence function; CMM, cardiometabolic multimorbidity; eGDR, estimated glucose disposal rate; TyG, triglyceride–glucose index.

Table S4. Cox models with outcome ascertainment restricted through 2018

| **Model** | **Index and contrast** | **Participants** | **CMM events** | **HR (95% CI)** | **P value** | **PH P (index)** | **PH P (global)** | **Holm-adjusted P** | **BH-adjusted P** |
| --- | --- | --- | --- | --- | --- | --- | --- | --- | --- |
| Model 3 | eGDR (per 1-SD lower) | 6,385 | 420 | 2.07 (1.88–2.28) | <0.001 | 0.834 | 0.647 | <0.001 | <0.001 |
| Model 3 | TyG (per 1-SD higher) | 6,385 | 420 | 1.73 (1.59–1.88) | <0.001 | 0.577 | 0.722 | <0.001 | <0.001 |
| Model 3 | AIP (per 1-SD higher) | 6,385 | 420 | 1.55 (1.41–1.71) | <0.001 | 0.696 | 0.620 | <0.001 | <0.001 |
| Model 4 | eGDR (per 1-SD lower) | 6,385 | 420 | 1.74 (1.55–1.94) | <0.001 | 0.868 | 0.740 | <0.001 | <0.001 |
| Model 4 | TyG (per 1-SD higher) | 6,385 | 420 | 1.48 (1.35–1.62) | <0.001 | 0.530 | 0.804 | <0.001 | <0.001 |
| Model 4 | AIP (per 1-SD higher) | 6,385 | 420 | 1.30 (1.17–1.44) | <0.001 | 0.748 | 0.680 | <0.001 | <0.001 |

*Note.* Outcome ascertainment used only the 2013, 2015, and 2018 follow-up surveys. The analysis included 6,385 participants and 420 incident CMM events; 22 participants without a determinate post-baseline CMM status through 2018 were excluded only from this sensitivity analysis. Model 3 was primary; Model 4 additionally adjusted for baseline CKM stage. PH P values are based on scaled Schoenfeld residuals and showed no evidence of nonproportionality. Holm and BH corrections covered the three indices within each model. Abbreviations: AIP, atherogenic index of plasma; BH, Benjamini–Hochberg; CI, confidence interval; CKM, cardiovascular–kidney–metabolic; CMM, cardiometabolic multimorbidity; eGDR, estimated glucose disposal rate; HR, hazard ratio; PH, proportional hazards; SD, standard deviation; TyG, triglyceride–glucose index.

Table S5. Baseline characteristics of participants included in and excluded from the final analytic cohort

| **Characteristic** | **Included in final analytic cohort (n = 6,407)** | **Excluded from final analytic cohort (n = 7,197)** | **Available n (included/excluded)** | **Absolute SMD** |
| --- | --- | --- | --- | --- |
| Age, years | 58.1 (8.5) | 57.4 (8.8) | 6,407/7,197 | 0.074 |
| Male | 3,041 (47.5) | 3,800 (52.8) | 6,407/7,197 | 0.107 |
| Rural residence | 4,218 (65.8) | 4,109 (57.1) | 6,407/7,197 | 0.180 |
| Married/partnered | 5,741 (89.6) | 6,407 (89.0) | 6,407/7,197 | 0.019 |
| Current smoking | 2,013 (31.4) | 2,074 (31.1) | 6,407/6,678 | 0.008 |
| Past-year alcohol use | 2,206 (34.4) | 2,612 (36.4) | 6,407/7,181 | 0.041 |
| BMI, kg/m² | 23.4 (3.7) | 23.2 (3.9) | 6,368/4,325 | 0.046 |
| Waist circumference, cm | 84.9 (9.8) | 82.3 (20.6) | 6,407/4,380 | 0.159 |
| SBP, mmHg | 128.4 (20.8) | 129.0 (21.2) | 6,407/4,357 | 0.030 |
| DBP, mmHg | 75.1 (12.0) | 75.6 (12.3) | 6,407/4,357 | 0.048 |
| Self-reported hypertension | 1,406 (21.9) | 1,461 (20.5) | 6,407/7,132 | 0.036 |
| Self-reported diabetes | 336 (5.2) | 323 (4.5) | 6,405/7,101 | 0.032 |
| Self-reported kidney disease | 308 (4.8) | 325 (4.5) | 6,398/7,169 | 0.013 |
| Education: low | 4,460 (69.6) | 4,450 (61.8) | 6,407/7,195 | 0.164 |
| Education: middle | 1,305 (20.4) | 1,620 (22.5) | 6,407/7,195 | 0.052 |
| Education: high | 642 (10.0) | 1,125 (15.6) | 6,407/7,195 | 0.169 |

*Note.* The comparison population comprised 13,604 baseline participants aged 45-79 years without baseline clinical CVD. Participants were classified according to whether they were included in (n = 6,407) or excluded from (n = 7,197) the final analytic cohort. Excluded participants were those not included because of nonfasting blood sampling or incomplete index data, indeterminate follow-up CMM status or CKM stage, or missing LDL-C. Values are mean (SD) or n (%). Available n is shown because baseline data completeness varied. Absolute SMDs were used descriptively; no hypothesis tests were performed. Among 6,416 participants remaining after the index, follow-up, and CKM-stage requirements had been applied, only 9 (0.14%) were excluded at the final complete-case step because of missing LDL-C; their mean age was 61.7 years, 2 (22.2%) were men, 8 (88.9%) had baseline diabetes, 6 (66.7%) were in CKM stage 3, and 2 (22.2%) developed CMM. Given the small number, these characteristics are descriptive only. For education, SMDs are reported for each category. Abbreviations: BMI, body mass index; CKM, cardiovascular-kidney-metabolic; CMM, cardiometabolic multimorbidity; CVD, cardiovascular disease; DBP, diastolic blood pressure; LDL-C, low-density lipoprotein cholesterol; SBP, systolic blood pressure; SMD, standardized mean difference.

Table S6. Pairwise Spearman correlations among eGDR, TyG, and AIP

| **Index** | **eGDR** | **TyG** | **AIP** |
| --- | --- | --- | --- |
| eGDR | 1.000 | −0.332 | −0.304 |
| TyG | −0.332 | 1.000 | 0.891 |
| AIP | −0.304 | 0.891 | 1.000 |

*Note.* Values are Spearman rank correlation coefficients calculated using the original index scales in the final complete-case cohort (n = 6,407). All two-sided P values were <0.001. Lower eGDR, but higher TyG and AIP, indicate a more adverse metabolic profile. Abbreviations: AIP, atherogenic index of plasma; eGDR, estimated glucose disposal rate; TyG, triglyceride–glucose index.

# 2 Supplementary figure

Figure S1. Competing-risk analyses of incident CMM

(A) Cause-specific Cox and Fine–Gray estimates per 1-SD lower eGDR or higher TyG/AIP in 6,407 participants. Horizontal lines denote 95% CIs. (B–D) Aalen–Johansen cumulative incidence curves by adverse-direction quartiles, with death before CMM treated as a competing event. Q1 and Q4 represent the least and most adverse quartiles, respectively; 9-year markers show estimates with 95% CIs. Gray’s P values compare the four curves. Abbreviations: AIP, atherogenic index of plasma; CI, confidence interval; CIF, cumulative incidence function; CMM, cardiometabolic multimorbidity; CS, cause-specific; eGDR, estimated glucose disposal rate; FG, Fine–Gray; HR, hazard ratio; SD, standard deviation; sHR, subdistribution hazard ratio; TyG, triglyceride–glucose index.
